# Is it time to shift the attention on early stages embryo development to avoid inconclusive evidence on HPV-related infertility: debate and proposal

**DOI:** 10.1186/1477-7827-12-48

**Published:** 2014-05-31

**Authors:** Marco Noventa, Alessandra Andrisani, Salvatore Gizzo, Giovanni B Nardelli, Guido Ambrosini

**Affiliations:** 1 Department of Woman and Child Health, University of Padua, Gynecologic and Obstetric Clinic, Giustiniani 3 street, 35128 Padua, Italy

**Keywords:** HPV infection, Human reproduction, Sperm infection, Couple infertility, Early miscarriage, Study BIAS, Embryo development, Blastocyst, Trophoblastic cells

## Abstract

**Background:**

Current evidence about *in-vivo* effects of HPV cannot definitively clarify the possible negative role of this worldwide common infection in early embryo development. However *in-vitro* evidence, seems to underline a possible negative effect of HPV in increasing blastocyst apoptosis and in reducing the endometrial implantation of trophoblastic cells. On these bases we believe that a new scientific approach is necessary to better understand the real role of male and female HPV infection in infertility and early pregnancy development.

**Methods:**

English literature review of manuscripts focused on HPV infection and human reproduction was conducted. We performed a critical analysis of evidence and possible bias affecting both *in-vivo* and *in-vitro* studies regarding this topic.

**Results:**

The biggest limitation of the *in-vivo* studies is due to the inappropriate timing of HPV effects evaluation since evidence about *in-vitro* studies strongly suggests that a large part of HPV negative effects occurs during a very early stage of embryo development. All the efforts of the scientific community to investigate the real role of HPV in human reproduction disorders cannot underestimate the severe BIAS of actual evidence in postulating new hypothesis and research projects which are fundamental to clarify if HPV may be associated with unexplained couples infertility and early miscarriages.

**Conclusions:**

If the relationship between HPV gametes infection and early human reproduction step impairment will be confirmed, the HPV male and couple vaccination may represent a reliable option to improve fertility in some couples affected by infertility actually classified as “idiopathic” but maybe linked to HPV infection.

## Background

Starting from our recent systematic literature review about “Male and Couple Fertility Impairment due to HPV-DNA Sperm Infection” we focused our attention on the controversies concerning HPV infection in human reproduction impairment [1]. Current evidence about *in-vivo* effects of HPV cannot definitively clarify the possible negative role of this worldwide common infection in early embryo development [2-6]. However, *in-vitro* evidence seems to underline a possible negative effect of HPV in increasing blastocyst apoptosis and in reducing the endometrial implantation of trophoblastic cells [7-15]. Despite Matovina et al. detected the HPV-DNA in miscarriages specimens without any significant association [16], in our knowledge only two clinical studies demonstrated a possible relationship between HPV and early pregnancy loss. Hermonat et al. comparing the prevalence of HPV in samples from spontaneous miscarriages and elective abortions found that 15 of the 25 spontaneous samples (60%) were positive for HPV-DNA sequences compared to only 3 of the 15 elective samples (20%) [2]. The higher percentage of HPV-DNA detection, if compared to voluntary abortions, lead the Authors to consider HPV as one of the possible etiologic agents responsible for early pregnancy loss. Perino et al., on a total of 199 couples undergoing to IVF cycles (*in-vitro fertilization*), found an increased risk of pregnancy loss both in case of male sperm infection (66.7% *versus* 15%) and of female cervical infection (40% *versus* 13.7%) [3].

Anyway the interesting results of Perino et al. should be interpreted with caution since several Authors reported approximately 24% of cervical HPV-DNA detection in women with ongoing pregnancy [17,18].

Other 3 available studies (other than Matovina et al. [16]) failed to find a possible correlation between HPV infection and early miscarriages [4-6]. Skoczynski et al., on a total of 129 women, found the HPV-DNA in 17.7% of spontaneous miscarriages samples and in 24.4% of placenta from term deliveries [4]. Also Conde-Ferraez et al., comparing 127 women with spontaneous abortion attending for curettage and 127 pregnant women at-term, found an HPV cervical prevalence of 24.4% and 15.2% respectively without significant correlation between HPV infection and pregnancy loss [5]. Ticconi et al., analyzing retrospectively 49 cases of women with unexplained recurrent miscarriages and 475 healthy controls, found a cervical HPV prevalence of 26.53% in cases (13 of 49) and 61.89% in controls (294 of 475) confirming the absence of a causal association between HPV and miscarriages [6].

## Discussion

We believe that all the *in-vivo* studies conducted till now (except for the perspective one of Perino et al.) are affected by different BIAS which probably led to confusion in considering the final results. The first possible BIAS is related to the HPV types detected: all the cited studies focused in particular on high-risk (Hr) genotype 16 and 18 extending the search only to few other Hr-genotypes or low risk HPV 6/11. The second BIAS is related to the sample detection methods of HPV presence which did not permit to rule out the possible contaminations derived by the passage through the genital tract. Adjunctively the study by Conde-Ferraez et al. and Ticconi et al., focusing only on cervical HPV detection, did not consider the possible BIAS related to the viral clearance (at time of cervical sample the patients may have already cleared the virus) which can lead to an underestimation of the problem [19]. The third BIAS is related to the fact that all these studies are built without considering the new inputs provided by recent *in-vitro* evidence which demonstrated that HPV sperm infection seems to have a possible role in adverse blastocyst development [7,14]. In particular it has been demonstrated that the spermatozoa is a vector for HPV transmission into fertilized oocytes and that the infected zygote (if the oocyte is fertilized) is able to perpetuate the viral genome expression at blastocyst stage and subsequently in trophoblastic cells [7,13,20].

On these bases we believe that a new scientific approach is necessary to better understand the real role of male and female HPV infection in early pregnancy development and infertility. We think that the recent *in-vitro* evidence on this field represents a good starting point to plan new perspective studies and to avoid the above mentioned BIAS.

Yet in 1996 Cabrera et al. in a murine experimental study demonstrated that spermatozoa carrying HPV-16 and −18 viral genome can be transfected into a mouse blastocyst with viral DNA localization both in the inner cells mass and trophoblastic cells [14]. The consequences of this possible “colonization” were subsequently evaluated by different Authors who confirmed the possible adverse effects of HPV at different steps of human embryo development. Calinisan et al. found that mouse blastocysts incubated with HPV-DNA-16 fragments showed an increased rate of DNA fragmentation and apoptosis [15]. This interesting result was subsequently confirmed and deepen by Henneberg et al. who defined the exact timing of HPV embryo impairment. In particular the Author showed how HPV-16 and −18 are able to inhibit the embryo development only at 2 cells stage (but not at 4–8 cells stage) with a peak percentage of about 30% after HPV-16 exposure and a major inhibition of blastocyst hatching process after HPV-18 exposure [10]. Different Authors demonstrated also the negative effect of HPV presence in trophoblastic cells which could result in abnormal placentation and maybe in early pregnancy loss. You et al. demonstrated that trophoblastic cells, like squamous epithelium, are broadly permissive for HPV showing some similarities in the gene expression repertoire between these two cells. She found an active viral genome expression (both early and late genes) in 3A trophoblastic cells previously cultured with HPV 16,18,11,31. Moreover she found, in different *in-vitro* studies, that HPV-31 infection and HPV-16 E6 and E7 oncogenes caused both a decrease in 3A trophoblastic cells number and a low trophoblast-endometrial cells adhesion in the first week after the exposure [12,13]. The evidence that HPV-16 is able to complete its life cycle in trophoblastic cells was previously reported by Liu et al. [21]. These findings provided to expand HPV biology, to support the hypothesis of a possible link between HPV and some spontaneous abortions, and to search a new technology for studying HPV.

These effects were also confirmed by Gomez et al. who found a 3- to 6- fold greater rate of apoptosis in trophoblastic cells transfected with a plasmid containing the entire HPV-16 genome and a progressive decrease of trophoblast invasion ability (25.2-57.6%) from day 3 until day 15 after transfection compared to negative controls [9]. Also Hong et al. showed a similar trend with a reduction both of implantation rate (less 37.2%) and of migratory/invasive activity in embryos exposed to HPV-16 [7].

The reasons which could explain the reduction of invasiveness of trophoblastic cells were suggested by Boulenouar et al. who found a down-regulation of E-cadherin (a fundamental protein for an adequate cell-to-cell adhesion) in trophoblastic cells expressing HPV-16 viral genome [8].

Although all the considered studies have important limitations (which should not be underestimated) linked to the in-vitro artificial and experimental conditions, most of the analyzed data suggested a possible adverse effect of HPV infection in early pregnancy development and maybe in idiopathic couple infertility. The demonstration that also the spermatozoa can act as a vector of HPV viral genome into oocyte [20] force the scientific community to understand what may be the clinical implication of this discovery. In fact, in 2010 Syrjanen reported that peri-conceptual transmission could theoretically occur via infected oocyte or spermatozoa [22]. So, although currently no studies exist on HPV detection in oocytes, theoretically, subsequent virus transmission might originate from the embryos soon after fertilization (such as via spermatozoa carrier) [20,22].

This fact acquires an even greater importance since the recent estimated prevalence of HPV sperm infection is about 10% (95% CI:7–14%) in general male population and 16% (95% CI:10–23%) in men affected by unexplained infertility [23].

So, focusing the investigations on HPV implications in human reproduction, it is mandatory to shift the older concept of infection with female gender target (i.e. oncologic risk in infected women respect to men) and to start in considering the HPV infection as a couple or, even better, a men problem.

In agreement with this new conception, it is intuitive that all the available *in-vivo* studies are not useful to completely understand the HPV exact role in human infertility because study methods, patients selection and idea conception seem to be inadequate.

The biggest limitation of the *in-vivo* studies is due to the inappropriate timing of HPV effects evaluation since evidence about *in-vitro* studies strongly suggests that a large part of HPV negative effects are carried out during a very early stage of embryo development (within the first two weeks after conception). These effects are very difficult to be demonstrated by clinical studies since it is possible to detect a causal-effects ratio on human reproduction only starting from the third week after conception (five week of amenorrhea). In this way all clinical studies lose a large part of negative effects demonstrated by *in-vitro* experimental studies.

Anyway clinical evidence can elucidate the consequences of HPV in late embryos development and pregnancy evolution. The detection of HPV in the placental tissue [24] lead to speculate about its possible role in adverse pregnancy outcomes since only few recent studies find an association between viral detection, placental abnormalities and preterm delivery [9,25,26]. Despite the increasing evidence of HPV vertical transmission [27,28], this route is regarded as less clinically important because of the detections of transient HPV-DNA. However, recent studies have provided clear evidence of papillomavirus productive infection in lymphocytes and placenta [29]. Furthermore, a model of papillomavirus latency has been recently proposed that could explain the failure or transience in HPV detection observed in some infected infants. This new evidence of hematogeneous and vertical spread of HPV suggests that these modes of transmission should be investigated in greater detail to obtain a better understanding of the infection and a fuller awareness of the preventive measures that can be taken against HPV-related diseases [30].

## Conclusions

In conclusion all the efforts of the scientific community to investigate the real role of HPV in human reproduction disorders cannot underestimate the severe BIAS of previous evidence in postulating new hypothesis and research projects. Even if the choice of more appropriate laboratory techniques (ISH rather than PCR) could partially solve the BIAS linked to potential contamination of specimen, shifting the attention on men and couples rather than only on women represent a first step to solve the dilemma. As suggestion to Clinicians interested in the topic we invite to focus the attention on idiopathic infertile couples undergoing ART cycles, theoretically the most appropriate cohort to plan both *in-vitro* and *in-vivo* further studies.

We must definitively understand if the hypothesized adverse effects of HPV in human reproduction is only a supposition derived from a “inconclusive” scientific speculation or if we are faced to a new important discovery which can potentially clarify the exact mechanism of some cases of unexplained couples infertility and of early miscarriages linked to HPV infection.

So If the suspected HPV role in early human reproduction impairment will be confirmed by well conducted further studies, the couple vaccination before attempting both spontaneous and assisted pregnancy could be a reliable and effective tool [31].

The rationale for proposing the use of HPV vaccination (Gardasil® or Cervarix®) in couple attempting ART cycle is based on the evidence that in man the HPV sperm infection seems associated with some spermatic parameters impairment [1] and in women HPV vaccination strength the immunological response to the HPV genotype already detected preventing de novo coinfection and superinfection by a different hr-HPV and lr-HPV genotypes [31]. As theoretical assumption, couple vaccination may potentially reduce some “undetected early miscarriage” actually erroneous considered as ART failure (lack of embryos implantation) increasing the pregnancy rate (first aim of ART procedures).

If in infertile couple the fertility rate will result increased after vaccination, this can be considered an indirect confirmation of our hypothesis and a suggestion to start more appropriate *in-vitro* and epidemiological studies on this field.

Improvement in the knowledge of HPV-DNA sperm infection mechanisms, timing, and link to fertility impairment may explain some of the actual “idiopathic” male and couple infertility.

## Competing interests

The authors declare that they have no competing interests.

## Authors' contributions

MN & SG carried out literature search, critical analysis of data and written the manuscript. AA carried out language revision and critical comment. GBN and GA participated in its design and coordination and helped to draft the manuscript. All authors read and approved the final manuscript.

## References

[B1] Gizzo S Ferrari B Noventa M Ferrari E Patrelli TS Gangemi M Nardelli GB Male and couple fertility impairment due to HPV-DNA sperm infection: update on molecular mechanism and clinical impact-systematic review Biomed Res Int 2014 2014 230263 24783196 10.1155/2014/230263 PMC3982419

[B2] Hermonat PL Han L Wendel PJ Quirk JG Stern S Lowery CL Rechtin TM Human papillomavirus is more prevalent in first trimester spontaneously aborted products of conception compared to elective specimens Virus Genes 1997 14 13 17 10.1023/A:1007975005433 9208451

[B3] Perino A Giovannelli L Schillaci R Ruvolo G Fiorentino FP Alimondi P Cefalù E Ammatuna P Human papillomavirus infection in couples undergoing in vitro fertilization procedures: impact on reproductive outcomes Fertil Steril 2011 95 1845 1848 10.1016/j.fertnstert.2010.11.047 21167483

[B4] Skoczyński M Goździcka-Józefiak A Kwaśniewska A Prevalence of human papillomavirus in spontaneously aborted products of conception Acta Obstet Gynecol Scand 2011 90 1402 1405 10.1111/j.1600-0412.2011.01189.x 21585342

[B5] Conde-Ferráez L Chan May Ade A Carrillo-Martínez JR Ayora-Talavera G González-Losa Mdel R Human papillomavirus infection and spontaneous abortion: a case–control study performed in Mexico Eur J Obstet Gynecol Reprod Biol 2013 170 468 473 10.1016/j.ejogrb.2013.07.002 23910697

[B6] Ticconi C Pietropolli A Fabbri G Capogna MV Perno CF Piccione E. Recurrent miscarriage and cervical human papillomavirus infection Am J Reprod Immunol 2013 70 343 346 24102829 10.1111/aji.12156

[B7] Hong LJ Oshiro BT Chan PJ HPV-16 exposed mouse embryos: a potential model for pregnancy wastage Arch Gynecol Obstet 2013 287 1093 1097 10.1007/s00404-013-2711-5 23307167

[B8] Boulenouar S Weyn C Van Noppen M Moussa Ali M Favre M Delvenne PO Bex F Noël A Englert Y Fontaine V Effects of HPV-16 E5, E6 and E7 proteins on survival, adhesion, migration and invasion of trophoblastic cells Carcinogenesis 2010 31 473 480 10.1093/carcin/bgp281 19917629

[B9] Gomez LM Ma Y Ho C McGrath CM Nelson DB Parry S Placental infection with human papillomavirus is associated with spontaneous preterm delivery Hum Reprod 2008 23 709 715 10.1093/humrep/dem404 18184644

[B10] Henneberg AA Patton WC Jacobson JD Chan PJ Human papilloma virus DNA exposure and embryo survival is stage-specific J Assist Reprod Genet 2006 23 255 259 10.1007/s10815-006-9030-8 16871451 PMC3506371

[B11] Weyn C Vanderwinden JM Rasschaert J Englert Y Fontaine V Regulation of human papillomavirus type 16 early gene expression in trophoblastic and cervical cells Virology 2011 412 146 155 10.1016/j.virol.2010.12.056 21276600

[B12] You H Liu Y Agrawal N Prasad CK Chiriva-Internati M Lowery CL Kay HH Hermonat PL Infection, replication, and cytopathology of human papillomavirus type 31 in trophoblasts Virology 2003 316 281 289 10.1016/j.virol.2003.08.020 14644610

[B13] You H Liu Y Agrawal N Prasad CK Edwards JL Osborne AF Korourian S Lowery CL Hermonat PL Multiple human papillomavirus types replicate in 3A trophoblasts Placenta 2008 29 30 38 10.1016/j.placenta.2007.08.005 17905430

[B14] Cabrera M Chan PJ Kalugdan TH King A Transfection of the inner cell mass and lack of a unique DNA sequence affecting the uptake of exogenous DNA by sperm as shown by dideoxy sequencing analogues J Assist Reprod Genet 1997 14 120 124 10.1007/BF02765781 9048243 PMC3454828

[B15] Calinisan JH Chan SR King A Chan PJ Human papillomavirus and blastocyst apoptosis J Assist Reprod Genet 2002 19 132 136 10.1023/A:1014736805127 12005308 PMC3468262

[B16] Matovina M Husnjak K Milutin N Ciglar S Grce M Possible role of bacterial and viral infections in miscarriages Fertil Steril 2004 81 662 669 10.1016/j.fertnstert.2003.08.020 15037417

[B17] Eppel W Worda C Frigo P Ulm M Kucera E Czerwenka K Human papillomavirus in the cervix and placenta Obstet Gynecol 2000 96 337 341 10.1016/S0029-7844(00)00953-4 10960622

[B18] Rombaldi RL Serafini EP Mandelli J Zimmermann E Losquiavo KP Transplacental transmission of Human Papillomavirus Virol J 2008 5 106 10.1186/1743-422X-5-106 18817577 PMC2567316

[B19] Saccardi C Gizzo S Noventa M Anis O Di Gangi S Patrelli TS D’Antona D Nardelli GB High-risk human papillomavirus DNA test: could it be useful in low-grade cervical lesion triage? Five-year follow-up Reprod Sci 2014 21 198 203 10.1177/1933719113492214 23744882

[B20] Foresta C Patassini C Bertoldo A Menegazzo M Francavilla F Barzon L Ferlin A Mechanism of human papillomavirus binding to human spermatozoa and fertilizing ability of infected spermatozoa PLoS One 2011 6 e15036 10.1371/journal.pone.0015036 21408100 PMC3051064

[B21] Liu Y You H Chiriva-Internati M Korourian S Lowery CL Carey MJ Smith CV Hermonat PL Display of complete life cycle of human papillomavirus type 16 in cultured placental trophoblasts Virology 2001 290 99 105 10.1006/viro.2001.1135 11887784

[B22] Syrjänen S Current concepts on human papillomavirus infections in children APMIS 2010 118 494 509 10.1111/j.1600-0463.2010.02620.x 20553530

[B23] Laprise C Trottier H Monnier P Coutlée F Mayrand MH Prevalence of human papillomaviruses in semen: a systematic review and meta-analysis Hum Reprod 2014 29 640 651 10.1093/humrep/det453 24365799

[B24] Weyn C Thomas D Jani J Guizani M Donner C Van Rysselberge M Hans C Bossens M Englert Y Fontaine V Evidence of human papillomavirus in the placenta J Infect Dis 2011 203 341 343 10.1093/infdis/jiq056 21208925 PMC3071105

[B25] Zuo Z Goel S Carter JE Association of cervical cytology and HPV DNA status during pregnancy with placental abnormalities and preterm birth Am J Clin Pathol 2011 136 260 265 10.1309/AJCP93JMIUEKRPIW 21757599

[B26] Kim YM Chaiworapongsa T Gomez R Bujold E Yoon BH Rotmensch S Thaler HT Romero R Failure of physiologic transformation of the spiral arteries in the placental bed in preterm premature rupture of membranes Am J Obstet Gynecol 2002 187 1137 1142 12439491 10.1067/mob.2002.127720

[B27] Hahn HS Kee MK Kim HJ Kim MY Kang YS Park JS Kim TJ Distribution of maternal and infant human papillomavirus: risk factors associated with vertical transmission Eur J Obstet Gynecol Reprod Biol 2013 169 202 206 10.1016/j.ejogrb.2013.02.024 23578811

[B28] Lee SM Park JS Norwitz ER Koo JN Oh IH Park JW Kim SM Kim YH Park CW Song YS Risk of vertical transmission of human papillomavirus throughout pregnancy: a prospective study PLoS One 2013 8 e66368 10.1371/journal.pone.0066368 23785495 PMC3681772

[B29] Foresta C Bertoldo A Garolla A Pizzol D Mason S Lenzi A De Toni L Human papillomavirus proteins are found in peripheral blood and semen Cd20+ and Cd56 + cells during Hpv-16 semen infection BMC Infect Dis 2013 13 593 10.1186/1471-2334-13-593 24341689 PMC3878630

[B30] Freitas AC Mariz FC Silva MA Jesus AL Human papillomavirus vertical transmission: review of current data Clin Infect Dis 2013 56 1451 1456 10.1093/cid/cit066 23392393

[B31] Gizzo S Noventa M Nardelli GB Gardasil administration to hr-HPV-positive women and their partners Trends Pharmacol Sci 2013 34 479 480 10.1016/j.tips.2013.07.001 23896431

